# Anti-seizure medication exposure and the risk of dementia: A meta-analysis of observational studies

**DOI:** 10.3389/fneur.2023.1133816

**Published:** 2023-03-22

**Authors:** Lei Zhang, Hai-yin Jiang, Wen-juan Liu

**Affiliations:** ^1^Psychosomatic Department, Hangzhou Traditional Chinese Medicine Hospital Affiliated to Zhejiang Chinese Medical University, Hangzhou, China; ^2^State Key Laboratory for Diagnosis and Treatment of Infectious Diseases, Collaborative Innovation Center for Diagnosis and Treatment of Infectious Diseases, The First Affiliated Hospital, College of Medicine, Zhejiang University, Hangzhou, China; ^3^Affiliated Mental Health Center & Hangzhou Seventh People's Hospital, Zhejiang University School of Medicine, Hangzhou, China

**Keywords:** anti-seizure, second generation, cognitive, systematic, meta-analysis

## Abstract

**Objective:**

There is growing evidence of a relationship between anti-seizure medication (ASM) use and the risk of dementia. This study examined this association using a meta-analysis approach.

**Methods:**

PubMed, EMBASE, and Cochrane Library were systematically searched for peer-reviewed observational studies published up to February 2023. Study quality was evaluated using the Newcastle-Ottawa Scale, and an overall odds ratio (OR) was pooled using fixed or random-effects models.

**Results:**

The analysis included 9 publications with 10 studies. The results showed that overall ASM exposure was associated with an increased risk of dementia [OR: 1.09, 95% confidence interval (CI): 1.03–1.15; *P* = 0.003] in general population. However, this association disappeared (OR: 1.02, 95% CI: 0.97–1.07; *P* = 0.361) when the study data adjusted for drug indications were pooled. Subgroup analysis based on individual drugs found only a positive association among those exposed to valproate, carbamazepine, and clonazepam. Furthermore, an increased risk was found in patients with bipolar disorder exposed to ASMs (OR: 1.43, 95% CI: 1.07–1.92; *P* = 0.015).

**Conclusions:**

The statistically significant association between ASM and dementia in general population may be driven by unmeasured confounding or several individual first-generation ASMs. However, a higher risk of dementia was observed among bipolar disorder patients treated with ASMs. Given the few included studies and evidence of high heterogeneity, further larger, prospective studies that control for important confounders are needed to verify our findings.

## 1. Introduction

Dementia is a progressive neurodegenerative disease characterized by progressive cognitive and functional decline constituting one of the leading causes of disability worldwide (1). It mainly affects older people, especially those over 65 years old (2). With the growing aging population, the number of people with dementia is predicted to triple to an estimated 152 million worldwide by 2050 (3). Considering the lack of treatment options, recognition of the risk factors of dementia may help to prevent the disease and could also inform appropriate interventions. Modifiable risk factors, including hypertension, infection, mental disorders, diabetes, and smoking, account for around 35% of dementia cases (4). Therefore, decreases in the incidence of dementia are partially attributable to avoiding some of these risk factors (5).

Anti-seizure medication (ASM) are widely used to treat epilepsy and bipolar disorder (6). While effective, they have been linked to negative clinical outcomes, such as increased risks of cognitive decline (7), cardiovascular disease (8), and fracture (9). Increasing numbers of epidemiological studies (10–18) have investigated the risk of dementia in ASM users; however, the results have been controversial. Some found an increased risk of dementia with ASM exposure, whereas others revealed no association. In the earliest cohort study, Carter et al. (10) reported that ASM use was associated with an increased risk of dementia; in three other large studies (14, 15, 18), however, dementia was not associated with ASM use. The findings of three studies (11, 12, 17) focusing on patients with bipolar disorder also conflicted. Because the various factors associated with ASM exposure (i.e., type of ASM and participants) may alter the risk of dementia differentially, these factors should be evaluated. Due to the increasing use of ASMs, determining the long-term effects of these drugs on dementia is important. The purpose of this systematic literature review and meta-analysis is to assess whether ASMs exposure increases the incidence of dementia.

## 2. Methods

Preferred Reporting Items for Systematic Reviews and Meta-analysis framework guidelines (PRISMA) were followed for this meta-analysis.

### 2.1. Data sources and search strategy

A comprehensive literature search of the PubMed, EMBASE, and Cochrane Library databases was conducted on February 2, 2023, according to the PRISMA statement, with no year restrictions. The search incorporated index terms (Mesh) and free text words for the search concepts: (antiepileptic AND antiseizure AND anticonvulsant AND valproic acid AND paraldehyde AND phenobarbitone AND levetiracetam AND lorazepam AND carbamazepine AND phenytoin AND midazolam AND lidocaine AND fosphenytoin AND bumetanide) AND (dementia OR Alzheimer OR frontotemporal dementia OR cognitive dysfunction OR cognitive impair OR cognitive decline OR vascular dementia OR multiinfarct dementia OR neurodegenerative diseases OR neurocognitive disorders) AND (risk OR ratio OR prospective studies OR epidemiologic studies OR case-control studies OR cohort studies). An additional search was conducted in the bibliographies of relevant articles and relevant reviews.

### 2.2. Selection criteria

The studies were assessed by two independent reviewers who determined whether the studies met the inclusion criteria. Observational studies were included if they were: (1) a peer-reviewed study with a case–control or cohort design published in English, (2) included ASM exposure preceding a diagnosis of dementia, (3) included participants 18 years or older, (4) explored the association between ASM exposure and the risk of dementia, and (5) provided sufficient data to allow the calculation of risk estimates if adjusted data were not provided. Case reports, case series, animal studies, editorials, reviews, and meta-analyses were excluded. Studies that considered dementia as comorbidity and not as an outcome were also excluded.

### 2.3. Data extraction

Two authors extracted information from all selected studies using piloted data extraction sheets. Any discrepancies in the extracted data were resolved by a third author. The following information was collected from each study: author, publication year, study location, sample demographics, information on ASM exposure, diagnostic criteria for dementia, number of subjects in each group, statistical adjustments, and study quality.

### 2.4. Risk of bias and quality assessment

The quality of the included observational studies was assessed using the Newcastle-Ottawa Scale (NOS) (19), which is recommended by the Cochrane Handbook for Systematic Reviews of Interventions. The assessment focuses on three major areas: the study population selection, the comparability between the two groups, and the ascertainment of exposure (for case-control studies) or the outcome of interest (for cohort studies).

### 2.5. Statistical analysis

We used the STATA ver.16.0 (StataCorp., College Station, TX, USA) to perform meta-analysis. A random-effects model was used to pool the odds ratios (ORs) and 95% confidence intervals (CI) of individual studies; such models are optimal in terms of allowing the results to be generalized because they can deal with potential heterogeneity (20). ORs were considered as approximations of relative risks (RRs) or hazard ratios (HRs) because the dementia outcome under study is rare in all populations and subgroups under review. Splitting one study into several estimates leads to substantially more weight being assigned to this study in the meta-analysis, especially in a random-effects model. Therefore, we used a fixed-effects model to produce a pooled OR if more than three estimates from one study were provided, and then included this pooled OR in the meta-analysis. The *I*^2^ statistic was used to assess between-study heterogeneity; The *I*^2^ values were classified into four groups: of 0–29%, 30–49%, 50–74%, and 75–100%, representing very low, low, medium, and high inconsistency, respectively (21). Funnel plots and Egger's test were used to test the presence of potential publication bias within this review (22, 23). All the statistical tests were bilateral, and *P*-values < 0.05 indicated considered significant.

## 3. Results

### 3.1. Search results

**Figure 1:**
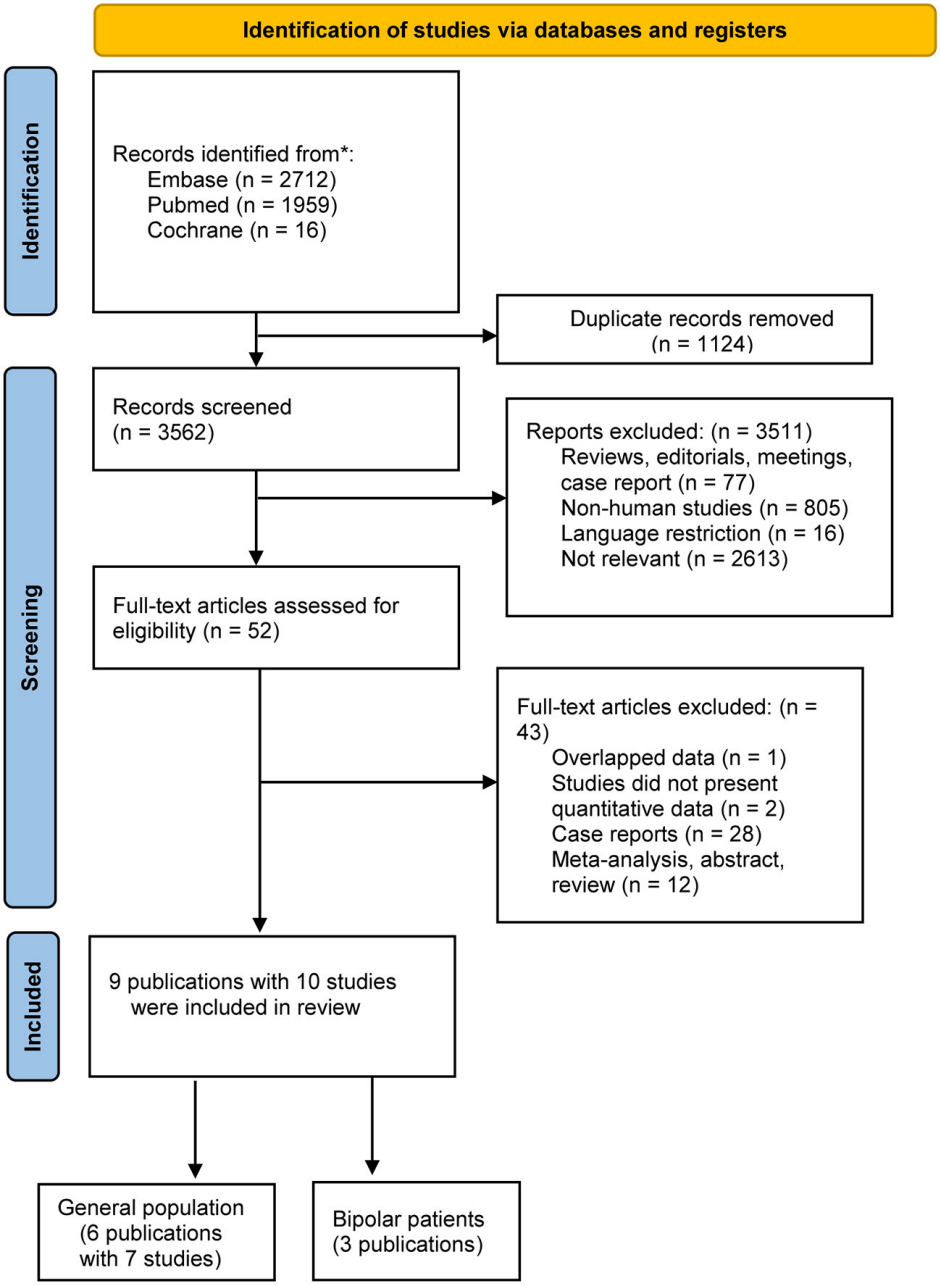
Flow chart of the search process and study selection.

After using the keywords, 4,687 records were identified in the initial search. Of these, 1,124 were duplicates, and 3,511 records were not relevant to the Research Topic after title and abstract screening, leaving 52 potentially eligible studies for which the full text was reviewed. Based on the inclusion and exclusion criteria, 9 publications with 10 studies were eligible for inclusion; all nine (10–18) were observational studies. Figure 1 is a flow diagram of the literature search and selection process.

### 3.2. Study characteristics

Table 1 summarizes the nine studies considered in this analysis. The studies included 1,629,213 participants from three different continents: five studies from Europe (13–16, 18), two from North America (10, 11), and two studies from Asia (12, 17). The publication year ranged from 2007 to 2022, and the sample sizes of the included studies ranged from 5,158 to 353,576. Exposure to ASMs was assessed using interviews or a drug prescription database. Three studies (11, 12, 17) assessed the use of ASMs and the development of dementia in individuals with bipolar disorder, and the remaining study evaluated this association in a general population. Regarding study quality, the mean NOS score for the nine studies was 8.3, indicating the high-quality of the included studies (Table 1). The score breakdown is given in Supplementary Tables S1, S2.

**Table 1:** Characteristics of the included studies.

**References**	**Location, setting**	**Study design**	**Age**	**Ascertainment of antiepileptic exposure**	**Outcome measurement**	**Number of participants**	**Confounding adjusted**	**Quality**
Carter et al. (10)	Canada, population-based	Cohort, general population	>65	Clinical examination or questionnaire	Modified mini-mental state examination or clinical examination	Exposed 67 Non-exposed 5,309	Age, sex, baseline 3MS score, head trauma, and stroke	6
Gerhard et al. (11)	USA, population-based	Cohort, patients with bipolar disorder	≥50	Pharmacy claims	ICD-9-CM	Exposed 20,778, Non-exposed 18,119	Gender, ethnicity, age, Medicaid eligibility, long-term care residency, depression, anxiety, alcohol-related disorders, drug-related disorders, arrhythmia, heart failure, myocardial infarction, other acute ischemic heart disease, other chronic ischemic heart disease, hypertension, cerebrovascular disease, diabetes mellitus, Parkinson's disease, antidepressant use, antipsychotic use, use of anti-anxiety medications	9
Tsai et al. (12)	Taiwan, population-based	Cohort, patients with bipolar disorder	≥20	Pharmacy claims	ICD-9-CM	Valproate exposed 1,792, Non-exposed 3,366	Age; sex; obesity; length of hospital admissions because of bipolar disorder; and the use of lithium, carbamazepine, antipsychotics, or benzodiazepine derivatives	9
Taipale et al. (13)	Finland, population-based	Case-control, general population	NA	Pharmacy claims	Hospital discharge register	Case 20,325, Control 81,300	Polypharmacy, stroke, depression, cardiovascular diseases, diabetes, and epilepsy	8
	German, population-based	Case-control, general population	≥60	Pharmacy claims	ICD-9	Case 70,718, Control 282,858	Polypharmacy, stroke, depression, cardiovascular diseases, diabetes, and epilepsy	8
Coupland et al. (14)	England, population-based	Case-control, general population	≥55	Pharmacy claims	Clinical codes or prescriptions	Case 58,769, Control 225,574	Body mass index, calculated as weight in kilograms divided by height in meters squared, smoking status, alcohol consumption, Townsend deprivation score, ethnic group, coronary heart disease, atrial fibrillation, heart failure, hypertension, hyperlipidemia, diabetes, stroke, transient ischemic attack, subarachnoid hemorrhage, renal disease, asthma, chronic obstructive pulmonary disease, anxiety, depression, bipolar disorder, schizophrenia, severe head injury, cognitive decline/memory loss, antihypertensive drugs, aspirin, hypnotics, anxiolytic drugs, non-steroidal anti-inflammatory drugs, statins, and with matching by age, sex, general practice, and calendar time	8
Jacob et al. (15)	German, population-based	Case-control, general population	≥60	Pharmacy claims	ICD-10	Case 50,575, Control 50,575	Epilepsy, hypertension, diabetes, hyperlipidemia, coronary heart disease, stroke including transient ischemic attack, intracranial injury, depression, bipolar disorder, mental and behavioral disorders due to use of alcohol, migraine, osteoporosis, prescription of benzodiazepines, prescription of antidepressants, and prescription of antipsychotics	8
Schnier et al. (16)	Wales, population-based	Cohort, general population	≥60	Pharmacy claims	ICD-10	563,151	Sex	6
Moon et al. (17)	Korean, population-based	Cohort, patients with bipolar disorder	≥50	Pharmacy claims	ICD-10	VPA exposed 1,785, Non-exposed 2,378	Diabetes, alcohol-related disorder, and use of anti-epileptics	7
Mur et al. (18)	UK, population-based	Cohort, general population	≥55	Pharmacy claims	Clinical codes or prescriptions	Case 2,124, Control 169,652	No	6

### 3.3. Meta-analysis

#### 3.3.1. Association between ASM use and dementia among general population

**Figure 2:**
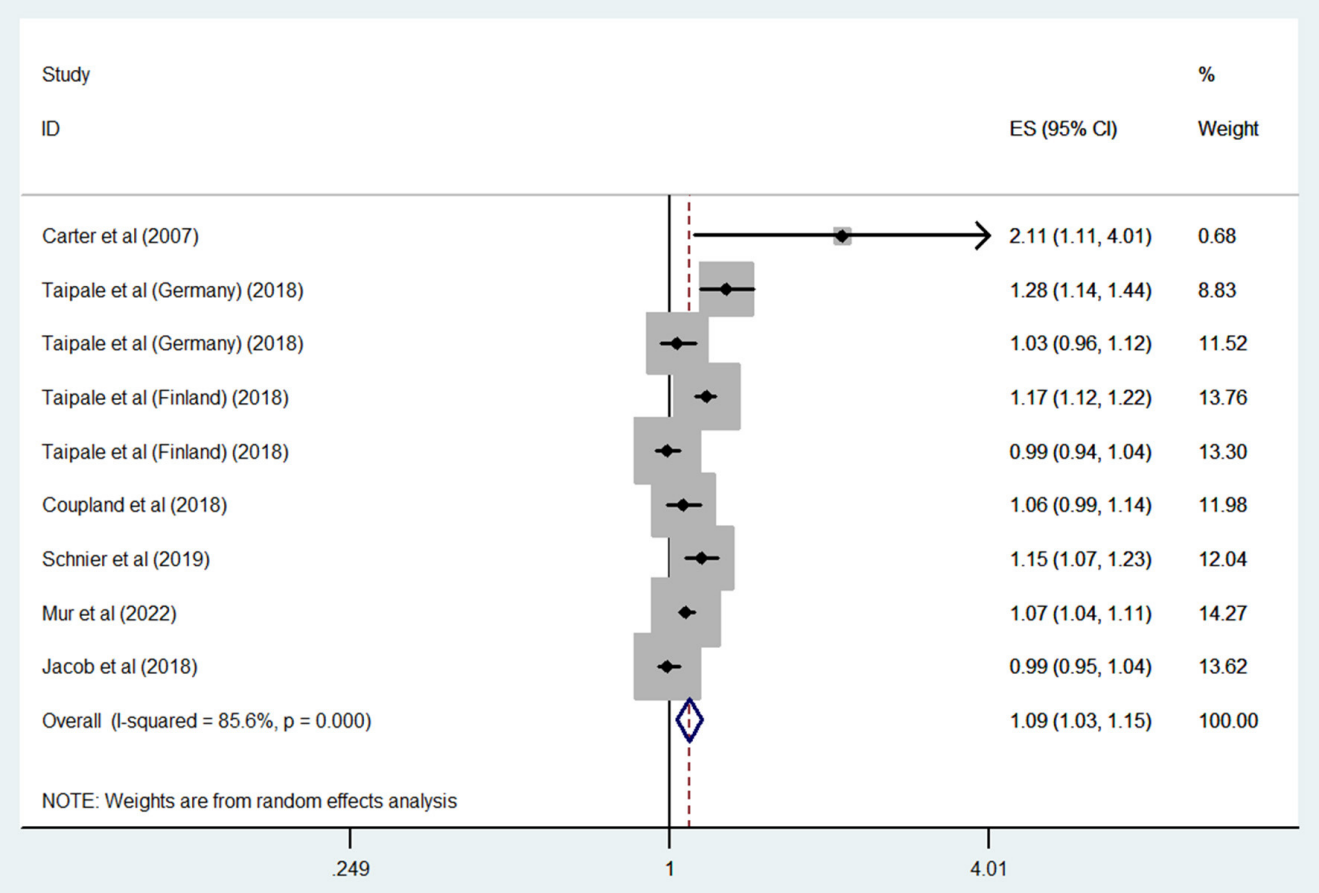
Forest plot of the overall risk of dementia in relation to ASMs use among the general population.

The results of all analyses are listed in Table 2. Six studies measured the relationship between overall ASM exposure and the risk of dementia among the general population. A meta-analysis of these studies with 9 estimates indicated that overall ASM exposure was significantly associated with an increased risk of dementia (OR: 1.09, 95% CI: 1.03–1.15; *P* = 0.003) (Figure 2). High heterogeneity was observed among these studies (*I*^2^ = 85.6%). As shown in Supplementary Figure S1, we did not find any evidence of publication bias (Begg's test, *P* = 0.3).

**Table 2:** Meta-analysis for studies included in the analysis.

**Subgroup analysis**	**Number of studies**	**Number of estimates**	**Pooled RR (95% CI), *I*^2^ statistics (%)**	**Model used**
General population	6	9	1.09 (1.03–1.15); *I*^2^ = 85.6%	Random effects
**Study design**				
Cohort	3	3	1.12 (1.02–1.23); *I*^2^ = 73.3%	Random effects
Case-control	3	6	1.07 (1–1.16); *I*^2^ = 89.3%	Random effects
**No. of adjustment variables**				
< 5	3	3	1.12 (1.02–1.23); *I*^2^ = 73.3%	Random effects
≥5	3	6	1.07 (1–1.16); *I*^2^ = 89.3%	Random effects
**Type of AEDs**				
Valproate	3	6	1.47 (1.29–1.67); *I*^2^ = 63.2%	Random effects
Carbamazepine	3	6	1.11 (1.03–1.19); *I*^2^ = 56.6%	Random effects
Clonazepam	3	6	1.21 (1.11–1.32); *I*^2^ = 29.2%	Random effects
Levetiracetam	2	2	1.25 (0.85–1.85); *I*^2^ = 80.9%	Random effects
Topiramate	2	2	1.11 (0.84–1.47); *I*^2^ = 0%	Random effects
Lamotrigine	2	2	1.05 (0.91–1.21); *I*^2^ = 0%	Random effects
Gabapentin	2	2	0.76 (0.49–1.18); *I*^2^ = 92.3%	Random effects
Pregabalin	2	2	0.84 (0.64–1.11); *I*^2^ = 73.1%	
Primidone	3	6	1.25 (0.95–1.64); *I*^2^ = 80.1%	Random effects
Phenytoin	2	5	1.05 (0.92–1.19); *I*^2^ = 49%	Random effects
Bipolar disorder	3	4	1.43 (1.07–1.92); *I*^2^ = 85.9%	Random effects
**Type of AEDs**				
Valproate	2	3	1.62 (1.38–1.89); *I*^2^ = 0%	Random effects

A subgroup analysis by study design found a significant association in cohort studies (OR: 1.12, 95% CI: 1.02–1.23; *P* = 0.02; *I*^2^ = 73.3%), but a non-significant trend toward an increased risk of dementia in case–control studies (OR: 1.07, 95% CI: 1–1.16; *P* = 0.059; *I*^2^ = 89.3%).

Considering the number of adjustment variables revealed a significantly increased dementia risk in those studies adjusting for fewer than five variables (OR: 1.12, 95% CI: 1.02–1.23; *P* = 0.02; *I*^2^ = 73.3%), but no significant association in those adjusting for more than five (OR: 1.07, 95% CI: 1–1.16; *P* = 0.059; *I*^2^ = 89.3%).

When we grouped studies by ASM type, significant associations were observed for those using valproate (OR: 1.47, 95% CI: 1.29–1.67; *P* < 0.001; *I*^2^ = 63.2%), carbamazepine (OR: 1.11, 95% CI: 1.03–1.19; *P* = 0.004; *I*^2^ = 56.6%), or clonazepam (OR: 1.21, 95% CI: 1.11–1.32; *P* < 0.001; *I*^2^ = 29.2%), but no significant association was observed for those using levetiracetam (OR: 1.25, 95% CI: 0.85–1.85; *P* = 0.253; *I*^2^ = 80.9%), topiramate (OR: 1.11, 95% CI: 0.84–1.47; *P* = 0.452; *I*^2^ = 0%), lamotrigine (OR: 1.05, 95% CI: 0.91–1.21; *P* = 0.527; *I*^2^ = 0%), gabapentin (OR: 0.76, 95% CI: 0.49–1.18; *P* = 0.225; *I*^2^ = 92.3%), pregabalin (OR: 0.84, 95% CI: 0.64–1.11; *P* = 0.227; *I*^2^ = 73.1%), primidone (OR: 1.25, 95% CI: 0.95–1.64; *P* = 0.11; *I*^2^ = 80.1%), or phenytoin (OR: 1.05, 95% CI: 0.92–1.19; *P* = 0.465; *I*^2^ = 32.3%).

#### 3.3.2. Association between ASM use and dementia among patients with bipolar disorder

**Figure 3:**
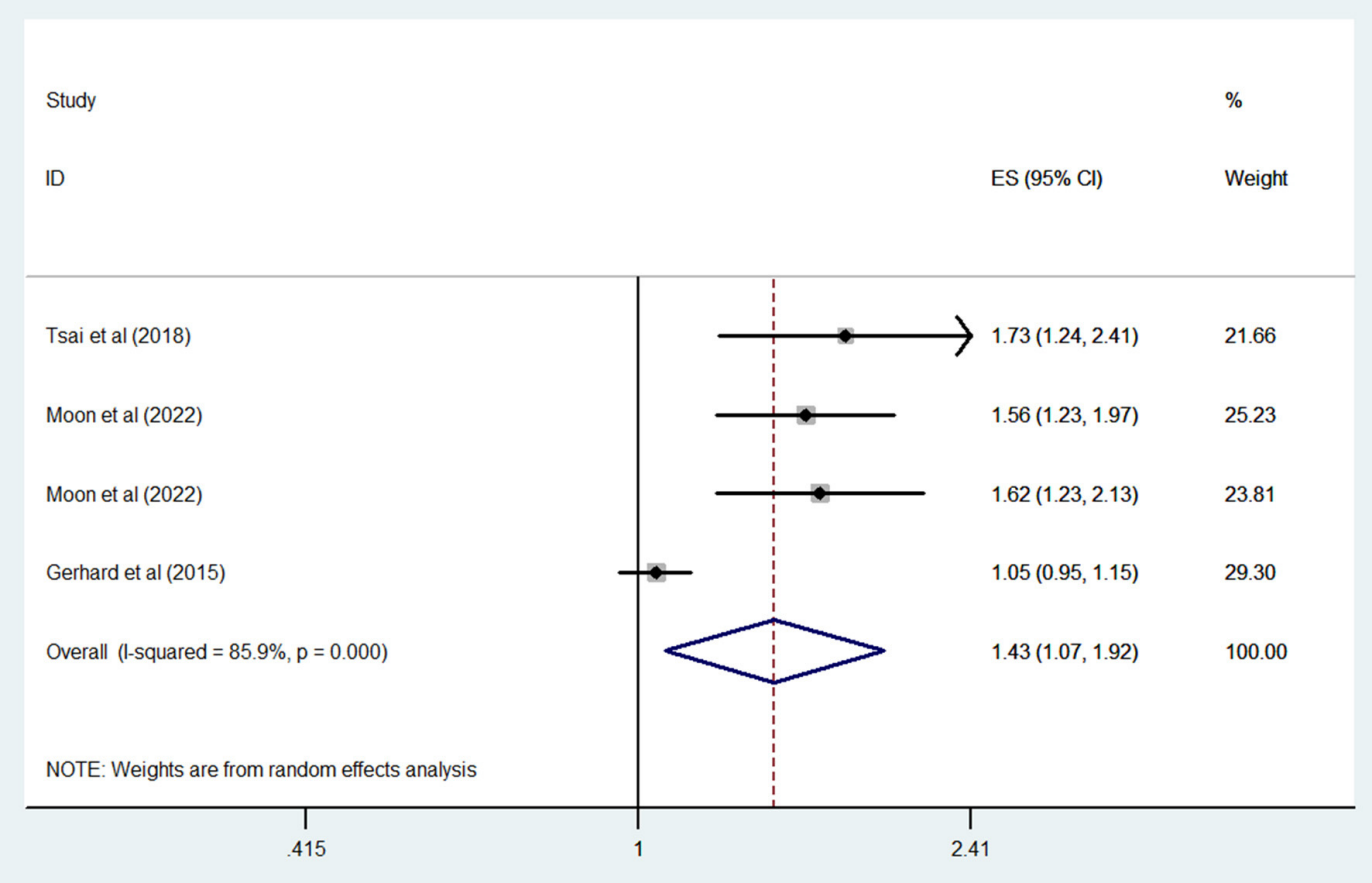
Forest plot of the overall risk of dementia in relation to ASMs use among patients with bipolar disorder.

Three studies compared the risk of dementia in bipolar disorder patients who were and were not exposed to ASMs; the combined OR of dementia was 1.43 (95% CI: 1.07–1.92; *P* = 0.015; *I*^2^ = 85.9%) (Figure 3). When our analysis limited to studies only evaluated valproate; the combined OR of dementia was 1.62 (95% CI: 1.38–1.89; *P* < 0.001; *I*^2^ = 0%).

## 4. Discussion

This meta-analysis of current observational evidence suggests that the statistically significant association between ASM use and dementia in general population can be partially explained by unmeasured confounding. However, subgroup analyses based on individual ASMs found that only valproate, carbamazepine, and clonazepam were associated with an increased risk of dementia. Furthermore, we found that bipolar disorder patients who were prescribed ASM showed an increased risk of dementia.

The impact of ASM use on cognitive function is controversial. Theoretically, ASMs can adversely affect cognitive functions by suppressing neuronal excitability or enhancing inhibitory neurotransmission (7, 24); however, several studies (25–27) have shown that exposure to several ASMs was associated with improved cognitive function because they also induce the neurogenesis of neural progenitor/stem cells both *in vitro* and *in vivo* (28). Consistent with the findings of these preclinical studies, the results of clinical studies that assessed the effects of ASMs on cognitive function or dementia varied. Furthermore, previous reviews (7, 24) have summarized this relationship, but failed to provide an overall estimate of the effects of ASMs on cognitive function or dementia. The authors noted that first-generation drugs had negative effects on cognitive function, but they were not found to increase the risk of dementia.

Although these modifying effects of ASMs on dementia are biologically plausible, the results of the included studies were discordant, as reflected in the high heterogeneity in the overall meta-analysis. This heterogeneity could not be accounted for in the subgroup analyses based on study design, location, or quality; number of adjustments; drug indications; and individual drugs. The existence of clinical heterogeneity should lead to a degree of statistical heterogeneity in the results.

Most of the studies in our overall analysis drew conclusions based on general-population data and did not consider the drug indications. However, epilepsy was shown to be associated with an increased risk of dementia (29). It is reasonable to speculate that this association may be overestimated if the studies did not adjust for this potential confounder. To minimize the effect of indication, we conducted a subgroup analysis based on the number of adjustment variables and found no significant association after we combined the estimates from the included studies adjusted for the drug indication. In addition to epilepsy, ASMs are commonly prescribed to treat bipolar disorder, depression, and other mental disorders (6). Previous meta-analysis have demonstrated that bipolar disorder is associated with an increased risk of dementia (30). Three included studies (11, 12, 17) focused on patients with bipolar disorder and used non-exposed patients as negative controls to minimize the effects of indication. In our meta-analysis, we observed an ~ 1.43-fold increase in the risk of dementia in patients with bipolar disorder who were exposed to ASMs.

The high heterogeneity of the overall analysis may also arise from the types of ASM. In our subgroup analysis of individual ASMs, only valproate, carbamazepine, and clonazepam, which are first-generation ASMs, were found to increase the risk of dementia. Previous studies demonstrated that the main cognitive effects of ASM use were impaired attention, vigilance, and psychomotor speed. One double-blind, placebo-controlled study (31) reported convincing evidence of improved motor skills after discontinuing valproate in patients with epilepsy. Two double-blind, placebo-controlled studies (32, 33) involving epilepsy patients on ASM monotherapy (mainly carbamazepine or valproate) observed that drug discontinuation significantly improved performance in tests that required complex cognitive processing under time pressure. However, most studies (25, 34, 35) tend to report little or no cognitive impairment associated with pregabalin or gabapentin in people with partial epilepsy. Consistent with the cognitive findings in epilepsy patients, our individual ASM analysis found that newer ASMs act more favorably on dementia risk compared with first-generation drugs. Recently, preclinical studies demonstrated a protective effect of levetiracetam on cognitive function. In the transgenic mice models of Alzheimer's disease, a low dose of levetiracetam could alleviate cognitive decline, through suppression of proinflammatory cytokines expression and inhibition of abnormal tau hyperphosphorylation (36, 37). In clinical study, levetiracetam improved performance on spatial memory and executive function tasks in patients with Alzheimer's disease (38). However, the beneficial role of levetiracetam on dementia was not detected in our analysis. Hence, our results of individual ASM on risk of dementia may be limited by sample size and need further investigation to clarify this issue.

To our knowledge, this meta-analysis is the first to explore the association between ASM use and dementia risk. The strengths of this work are the comprehensive search and the rigorous systematic review and meta-analysis of all relevant reports to date. We also performed several additional analyses to test the robustness of the results. Nonetheless, there are several limitations to this meta-analysis. First, residual confounders are always a concern in epidemiological observational studies. Second, all of the included studies considered Western populations and not subjects from Asia or Africa, which may have affected the generalizability of our results. Third, information on the dose of ASM used in the included studies could not be extracted; therefore, any exposure parameter possibly associated with dementia could not be defined.

In summary, this systematic review and meta-analysis only observed a greater risk of dementia with the use of valproate, carbamazepine, or clonazepam in general population. We also found that ASMs are associated with an increased risk of dementia in bipolar disorder. However, large, well-designed, prospective cohort studies that consider a greater number of confounding factors are warranted to verify our findings.

## Data availability statement

The original contributions presented in the study are included in the article/Supplementary material, further inquiries can be directed to the corresponding author.

## Author contributions

LZ and H-yJ searched the library, wrote the manuscript text, extracted data, and reviewed all articles. W-jL designed the manuscript. All authors reviewed the manuscript. All authors contributed to the article and approved the submitted version.
