# Supplementary material for: Anti-seizure medication exposure and the risk of dementia: A meta-analysis of observational studies
Source: Front Neurol. 2023 Mar 22;14:1133816. doi: 10.3389/fneur.2023.1133816 (PMC10073491; doi:10.3389/fneur.2023.1133816)

| **Table S1**  NOS for Assessment of Quality of Included Studies: Cohort Studies | | | | | | | | |
| --- | --- | --- | --- | --- | --- | --- | --- | --- |
| Study | Selection | | | | Comparability | | Outcomes | |
|  | Representativeness of exposed cohort? | Selection of the nonexposed cohort? | Ascertainment of exposure? | Demonstration that outcome of interest was not represent at the start of the study | Comparability of Cohort | Assessment of outcome | Was follow-up long enough for outcomes to occur | Adequacy of follow up of cohorts |
| Carter et al, 2007 | ★ | ★ | — | ★ | ★ | ★ | — | ★ |
| Gerhard et al, 2015 | ★ | ★ | ★ | ★ | ★★ | ★ | ★ | ★ |
| Tsai et al, 2018 | ★ | ★ | ★ | ★ | ★★ | ★ | ★ | ★ |
| Schnier et al, 2019 | ★ | ★ | ★ | ★ | — | ★ | — | ★ |
| Moon et al, 2019 | ★ | ★ | ★ | ★ | ★ | ★ | ★ | ★ |
| Mur et al, 2020 | ★ | ★ | ★ | — | — | ★ | ★ | ★ |

| **Table S2** NOS for Assessment of Quality of Included Studies: Case-Control Studies | | | | | | | | | |
| --- | --- | --- | --- | --- | --- | --- | --- | --- | --- |
| Study | Selection | | | | Comparability | | Exposure | | |
|  | Is the case definition adequate | Representativeness of cases | Selection of controls | Definition of controls | Study controls for age/sex | Study controls for at least 3 additional factors | Ascertainment of exposure | Same method of ascertainment of exposure | Nonresponse rate |
| Taipale et al, 2018 | ★ | ★ | ★ | ★ | ★ | ★ | ★ | ★ | — |
| Coupland et al, 2018 | ★ | ★ | ★ | ★ | ★ | ★ | ★ | ★ | — |
| Jacob et al, 2018 | ★ | ★ | ★ | ★ | ★ | ★ | ★ | ★ | — |

**Figure S1** The Begg funnel plot and Egger test for identifying publication bias in a meta-analysis of observational studies


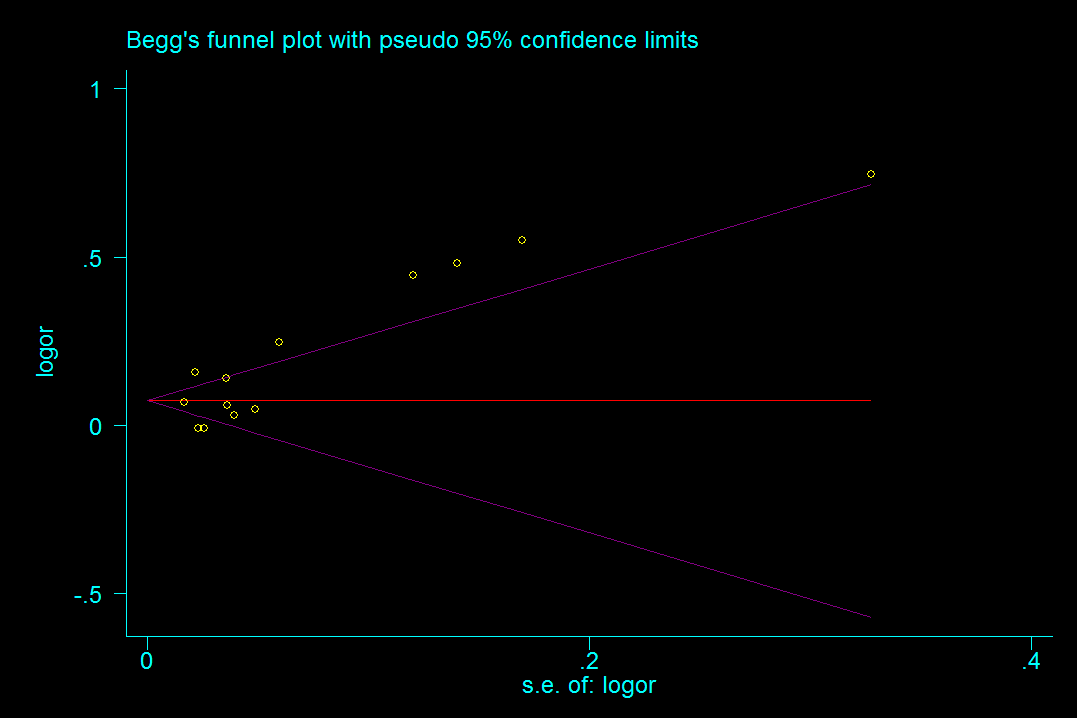

Supplement: Supplementary file 1 [file Table_1.DOCX]
